# Supplementary material for: A Geometric-Structure Theory for Maximally Random Jammed Packings
Source: Sci Rep. 2015 Nov 16;5:16722. doi: 10.1038/srep16722 (PMC4644945; doi:10.1038/srep16722)
Supplement: Supplementary Information [file srep16722-s1.pdf]

Supplementary Information:

A Geometric-Structure Theory for Maximally Random Jammed Packings

Jianxiang Tian[1;2], Yaopengxiao Xu[3], Yang Jiao[3], & Salvatore Torquato[4;5;6]

1 Department of Physics, Qufu Normal University, Qufu 273165, China

2 Department of Physics, Dalian University of Technology, Dalian 116024, China

3 Materials Science and Engineering, Arizona State University, Tempe Arizona 85287, USA

4 Department of Chemistry, Princeton University, Princeton New Jersey 08544, USA

5 Department of Physics, Princeton University, Princeton New Jersey 08544, USA

6 Program in Applied and Computational Mathematics, Princeton University, Princeton New Jersey, 08544, USA

In the Fig 5 of the main paper, we have compared the numerically obtained MRJ packing density φ_{MRJ} and the corresponding predicted value for superdisks with different deformation parameter *p*, small particle concentration *x*, and small-to-large size ratio *α.* Here the values of φ_{MRJ} for generating Fig. 5 are provided.

Table 1: Numerically obtained MRJ packing densities for superdisks with different deformation parameter *p*, small particle concentration *x*, and small-to-large size ratio *α.*

x=0.2	p=0.85	p=0.9	p=1.0	p=1.2	p=1.5	p=1.8	p=2.0	p=2.5	p=3.0
α=0.4	0.8697	0.8626	0.8485	0.871	0.8865	0.8955	0.9	0.9094	0.9154
α =0.6	0.8675	0.8613	0.8468	0.8685	0.8835	0.8964	0.9000	0.9105	0.9148
α =0.8	0.8643	0.8585	0.8496	0.8655	0.8832	0.8963	0.9030	0.9158	0.9227
α =1.0	0.8979	0.8858	0.8677	0.8921	0.9005	0.9118	0.9207	0.9294	0.9404

x=0.4	p=0.85	p=0.9	p=1.0	p=1.2	p=1.5	p=1.8	p=2.0	p=2.5	p=3.0
α =0.4	0.8727	0.8664	0.8531	0.8741	0.8878	0.8968	0.8998	0.9056	0.9106
α =0.6	0.8662	0.8606	0.8484	0.868	0.8843	0.8939	0.8987	0.9057	0.9103
α =0.8	0.8620	0.8554	0.8433	0.8622	0.8810	0.8967	0.9030	0.9130	0.9215
α =1.0	0.8866	0.8832	0.8716	0.8874	0.8995	0.9109	0.9188	0.9290	0.9448

x=0.6	p=0.85	p=0.9	p=1.0	p=1.2	p=1.5	p=1.8	p=2.0	p=2.5	p=3.0
α =0.4	0.8795	0.8722	0.8600	0.8797	0.8917	0.8993	0.9015	0.9077	0.9110
α =0.6	0.8675	0.8611	0.8486	0.8689	0.8847	0.8951	0.8998	0.9055	0.9093
α =0.8	0.8628	0.8559	0.8426	0.8633	0.8819	0.8955	0.9024	0.9150	0.9183
α =1.0	0.8962	0.8869	0.8725	0.8927	0.90315	0.9093	0.9164	0.9314	0.9388

x=0.8	p=0.85	p=0.9	p=1.0	p=1.2	p=1.5	p=1.8	p=2.0	p=2.5	p=3.0
α =0.4	0.8805	0.8738	0.8625	0.8808	0.8939	0.9020	0.9058	0.9140	0.9173
α =0.6	0.8670	0.8598	0.8485	0.8688	0.8854	0.8962	0.9014	0.9112	0.9147
α =0.8	0.8663	0.8578	0.8438	0.8664	0.8855	0.8984	0.9051	0.9164	0.9217
α =1.0	0.8845	0.8922	0.8683	0.8851	0.8992	0.9118	0.9172	0.9389	0.9387

Table 2: Predicted MRJ packing densities for superdisks with different deformation parameter *p*, small particle concentration *x*, and small-to-large size ratio *α*.

x=0.2	p=0.85	p=0.9	p=1.0	p=1.2	p=1.5	p=1.8	p=2.0	p=2.5	p=3.0
α =0.4	0.8675	0.8614	0.8385	0.8736	0.8961	0.9145	0.9189	0.9203	0.9239
α =0.6	0.8626	0.8564	0.8368	0.8680	0.8896	0.9076	0.9131	0.9159	0.9189
α =0.8	0.8602	0.8542	0.8396	0.8655	0.8872	0.9075	0.9128	0.9151	0.9165
alpha=1	0.8597	0.8538	0.8427	0.8667	0.8891	0.9094	0.9146	0.9160	0.9182

x=0.4	p=0.85	p=0.9	p=1.0	p=1.2	p=1.5	p=1.8	p=2.0	p=2.5	p=3.0
alpha=0.4	0.8754	0.8690	0.8431	0.8805	0.9031	0.9196	0.9233	0.9265	0.9296
alpha=0.6	0.8655	0.8591	0.8384	0.8694	0.8901	0.9058	0.9117	0.9148	0.9196
alpha=0.8	0.8607	0.8546	0.8333	0.8643	0.8853	0.9055	0.911	0.9149	0.9172
alpha=1	0.8597	0.8538	0.8416	0.8667	0.8891	0.9094	0.9146	0.9160	0.9182

x=0.6	p=0.85	p=0.9	p=1.0	p=1.2	p=1.5	p=1.8	p=2.0	p=2.5	p=3.0
alpha=0.4	0.8832	0.8767	0.8500	0.8874	0.9101	0.9246	0.9277	0.9318	0.9353
alpha=0.6	0.8685	0.8618	0.8386	0.8708	0.8907	0.9040	0.9103	0.9157	0.9203
alpha=0.8	0.8613	0.8550	0.8326	0.8631	0.8834	0.9036	0.9091	0.9132	0.9173
alpha=1	0.8597	0.8538	0.8416	0.8667	0.8891	0.9094	0.9146	0.9160	0.9182

x=0.8	p=0.85	p=0.9	p=1.0	p=1.2	p=1.5	p=1.8	p=2.0	p=2.5	p=3.0
alpha=0.4	0.8911	0.8843	0.8525	0.8944	0.9171	0.9297	0.9320	0.9381	0.9411
alpha=0.6	0.8714	0.8645	0.8385	0.8722	0.8912	0.9022	0.9088	0.9166	0.9210
alpha=0.8	0.8618	0.8555	0.8338	0.8620	0.8815	0.9016	0.9073	0.9115	0.9182
alpha=1	0.8597	0.8538	0.8416	0.8667	0.8891	0.9094	0.9146	0.9160	0.9182
